# Improving Patient Engagement in Psychological Interventions

**DOI:** 10.1192/bjo.2022.417

**Published:** 2022-06-20

**Authors:** Rajeswari Sivaji, RavindraB Belgamwar

**Affiliations:** North Staffordshire Combined Healthcare NHS trust, Stoke-on-Trent, United Kingdom

## Abstract

**Aims:**

The aim of this service evaluation project is to gain understanding about the reasons for service user's disengagement in psychological interventions. We felt that the findings of this project will enable services to better understand the experience of service users and help recognise why someone requesting services does not follow through. Around 68% of patients who were referred to psychological therapy did not complete therapy in our community mental health team highlighting a need to improve patient engagement in psychological interventions. Patients under secondary mental health services have complex needs and any referral decision to the most appropriate psychological intervention will need to be carefully considered as a part of their treatment plan. Premature termination from psychological interventions can lead to poor treatment outcome, waste staff time and contribute to unnecessary long waiting lists.

**Methods:**

Random sample of 20 service users who disengaged from psychological therapy were chosen and telephone interviews were conducted to determine their perspectives on reasons for their termination. Introductory letter informing about this project was posted to the service users and they were contacted after a week to gather information. The following themes were included in the interview questionnaire like demographic characteristics, psychopathological difficulties, problems related to therapy or therapist, external circumstantial problems, internal factors and service user views on satisfaction/achievement of therapy goals.

**Results:**

The results showed:

1. The most frequent reported reason for disengagement from psychological intervention was COVID-19 and internal factors (thinking that therapy would not help, low mood/too anxious, previous bad experience with therapy and feeling unwilling to open).
2. Number of session's service users attended ranged from 0 to 6 and no one completed the therapy.
3. Waiting time (from referral to start of therapy) ranged from 2 to 6 months.
4. 37.5% of service users were not aware about therapy details.

**Conclusion:**

The results were shared with staff via local meetings Recommendations were drawn to improve patient engagement and retention in therapy.

1. Outpatient pack resources developed to offer service users at appointments which has written information sheets about presenting problems, overview of psychological interventions/assessment and diaries for service users.
2. New template was drafted to improve the referral process to psychology by referrers having access to guides on how to assess a person's psychological needs, readiness for therapy and the provision of consultation slots with psychologists.

